# A Flexible All-Solid-State Asymmetric Supercapacitor Based on a Nanocomposite of Vanadium Oxide/Graphene and Polyaniline Hydrogel with Excellent Operational Stability and Energy Density

**DOI:** 10.1021/acsomega.5c03646

**Published:** 2025-09-15

**Authors:** Mohammad Barazandeh, Sayed Habib Kazemi, Farzad Roohi, Dawod S. Haydar, Inger Odnevall

**Affiliations:** a Department of Chemistry, 113403 Institute for Advanced Studies in Basic Sciences (IASBS), 45137-66731 Zanjan, Iran; b Department of Chemistry, College of Science, Salahaddin University—Erbil, 44002 Erbil, Kurdistan Region, Iraq; c KTH Royal Institute of Technology, Dep. Chemistry, Div. Surface and Corrosion Science, SE-100 44 Stockholm, Sweden

## Abstract

Porous polymer-based materials of high electrical conductivity are essential for a wide range of applications. An innovative nanocomposite of V_2_O_5_/rGO/polyaniline hydrogel (VG/PANi-HG) was synthesized using in situ oxidative polymerization in the presence of V_2_O_5_/rGO (VG) and phytic acid on a carbon fiber substrate. Subsequently, the corresponding electrode was employed as a flexible free-standing electrode and examined through a range of electrochemical methods to assess its performance in high-rate supercapacitors. The VG/PANi-HG supercapacitor demonstrated an excellent specific capacitance of 982 F g^–1^ at a current density of 2.7 A g^–1^. Furthermore, an asymmetric all-solid-state (ASC) device was assembled using VG/PANi-HG as a positive electrode in combination with a graphene oxide electrode as a negative electrode and a polyvinyl alcohol (PVA) hydrogel electrolyte. This flexible asymmetric device exhibited an impressive cycle life stability and a power density of 2 kW kg^–1^ at an energy density of 29.44 Wh kg^–1^. Our results reveal that the VG/PANi-HG electrode material is a promising candidate for future electrochemical energy storage devices.

## Graphical abstract

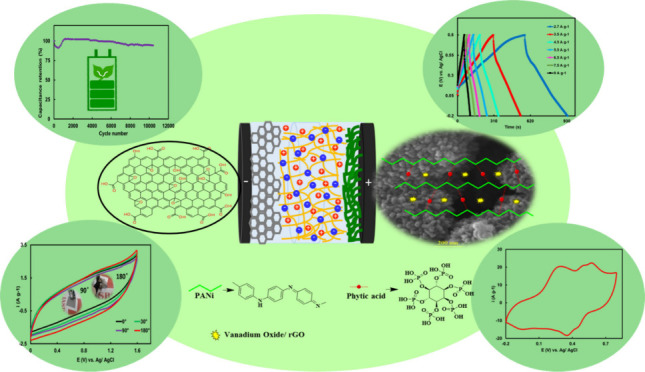

## 1 Introduction

To meet the increasing demand for modern electronic devices such as stretchable integrated circuits, laptops, phones and tablets, digital cameras and touch screens, it is crucial to develop light, thin, flexible, environmentally friendly, and portable energy storage devices of supercapacitors (SCs) and batteries.^1–3^ Supercapacitors have recently received more intense attention than batteries due to their unique properties including their fast charge–discharge characteristics, operational long-term cycle stability, and high-power densities.^4–6^ Electrochemical capacitors have demonstrated potential applications in energy management, hybrid electric vehicles, memory backup devices, military equipment, in addition to portable and wearable electronic systems.^7–9^ Electrical double-layer capacitors (EDLCs) and pseudocapacitors made of transition metal oxides and/or conducting polymers with electrical energy storage abilities through reversible electrochemical redox processes are two main classes of SCs based on the chemical and electrochemical behavior of their electrode materials.^9–16^ Due to their highly tunable electrochemical and electrical properties, conducting polymers (CPs), such as polypyrrole (PPy), polyaniline (PANi), and poly­(3,4-ethylene­dioxythiophene) (PEDOT) have attracted intense attention.^17–20^ Theoretically, fabrication of CPs into hydrogels can offer reasonable solutions for lightweight materials with considerable qualities from a large surface area, soft and biocompatible surfaces, and high permeability to electrolyte perspective.^21–23^ For applications where both permeability and electric conductivity are required simultaneously, CP hydrogels can therefore be considered as efficient alternatives for high-performance devices.^24–26^ However, the lack of reliable methods to design and fabricate CP hydrogels currently severely restricts their use. The porous 3-D hierarchical structure of the CP hydrogels allows quick electron transfer and ion diffusion throughout its entire network, resulting in the amplified electrical and electrochemical performance of the supercapacitor.^27–30^ Due to its high pseudocapacitance, high conductivity, controllable morphology, inherent flexibility, and lightweight properties, polyaniline (PANi) is one of the most important conducting polymers for charge storage purposes.^24,31–34^

Polyaniline hydrogels (PANi-HGs) can be considered for a wide variety of applications including biological and chemical sensors, electromagnetic shielding, and electrochemical energy storage devices.^33,35–37^ However, it has been reported that upon charging and discharging operations, PANi-HGs display mechanical deterioration and volumetric changes, which have a negative impact on their rate capability and cycle stability characteristics.^38,39^ To overcome these drawbacks, it has been suggested that CPs should be combined with carbon nanotubes, graphene, or transition metal oxides to create composite electrodes of enhanced electrochemical properties. Such integrations improve remarkably the mechanical integrity and stability of the CPs and hence the performance of the supercapacitor.^32,40,41^

Vanadium (V) oxides have been broadly applied as electrode materials in supercapacitors due to their high electrochemical activity and strong electron–electron interactions.^42,43^ Due to the various oxidation states of V (+II, + III, +IV, +V), the theoretical specific capacitance of vanadium oxides is significantly higher than for most other metal oxides.^44–46^ The synergistic effect of polyaniline, vanadium oxide and graphene oxide composites has previously been investigated and confirmed for supercapacitor applications, e.g., Tabrizi et al.[Bibr ref47] 2017, Yasoda et al.[Bibr ref48] 2019, and Viswanathan et al.[Bibr ref49] 2019.

The aim of this study was to generate an innovative hydrogel-based nanocomposite for future use as an electrode material in energy storage systems with increased specific capacitance and cycling stability. This was accomplished by synthesizing V_2_O_5_-reduced graphene oxide in a polyaniline hydrogel (VG/PANi-HG). The research hypothesis was that this approach would result in a synergistic effect between vanadium oxide-reduced graphene oxide and conductive hydrogel network and thereby create a supercapacitor electrode material of superior specific capacitance and energy density. Reduced graphene oxide (rGO) was used within the VG/PANi-HG nanocomposite to benefit from its high specific surface area, high power density, long cycle life, and excellent mechanical properties, thereby increasing the active surface area of the nanocomposite and the physical stability of the vanadium oxide nanoparticles.

The hydrogel fabrication was carried out directly on a carbon fiber substrate (binder-free). The charge storage capacity of the synthesized VG/PANi-HG nanocomposite as the positive electrode in an assembled flexible asymmetric supercapacitor, combined with a negative graphene oxide electrode (GO/CF), was investigated, and its performance was elucidated.

## 2 Results and Discussion

### 2.1 Multianalytical Characterization of the Synthesized Nanocomposite

The synthesized nanocomposite of V_2_O_5_-rGO (VG) and the polyaniline hydrogel (PANi-HG) was characterized using FTIR spectroscopy to assess the presence of surface functional groups, as shown in Figure 1a. The bands at 1570 and 1480 cm^–1^ were attributed to the stretching vibrations of the quinoid rings and the stretching vibrations of the benzenoid ring, respectively. Stretching vibrations of the C–N of secondary amines present in the PANi chains could be observed at approximately 1290 cm^–1^, and a band at 1240 cm^–1^ was assigned to the stretching mode of C=N. The band at 1070 cm^–1^ was related to the bending vibrations of the C–H bonds and the corresponding band related to the stretching vibrations of V=O was observed at 1004 cm^–1^. The symmetric and asymmetric stretching vibrations of V–O–V peaks were observed at approximately 520 and 736 cm^–1^, respectively.^48,50,51^ The FTIR spectrum of pure graphene oxide compared to reduced graphene oxide/vanadium oxide is presented in Figure S1.

The results of the XRD analysis acquired for the VG/PANi-HG composite and the pure hydrogel are illustrated as Figure 1b. The XRD patterns for the pure PANi hydrogel matched with the standard pattern for PANi (JCPDS 053-1890)^52^ with major peaks at almost 14°, 21°, and 25° corresponding to the (011), (113), and (200) crystal planes, respectively. In addition, the VG/PANi-HG nanocomposite spectrum revealed peaks (low (l), medium (m), strong (s) intensity) at 7.9° (l), 15.6° (l), 20.4° (s), 21.9° (l), 26.3° (s), 31.2° (s), 32.9° (l), 34.3° (l), 47.9° (l) and 51.6° (l) and 54.8° which may be indexed to (003), (002), (001), (011), (110), (040), (012), (130), (201), and (200) planes, respectively of the polyaniline structure and an orthorhombic crystal structure of vanadium oxide (JCPDS 001-0359 and 72-0433).^51,53–55^ The crystal size was calculated using the Debye–Scherer equation at 2θ = 25° for both cases (pure PANi hydrogel and VG/PANi-HG). The FWHM values for the pure hydrogel and composite were 0.38° and 0.48°, respectively. The calculated crystallite sizes were approximately 168.36 nm for the pure PANi hydrogel and 224.48 nm for VG/PANi-HG.

The chemical structure, elemental composition, and valence states of the outermost surface of the nanocomposite hydrogel were investigated by means of X-ray photoelectron spectroscopy (XPS). The results are presented in Figure 1c–f and Figure S2. The fitted C 1s spectrum (Figure 1c) shows three peaks at binding energies of 284.8 eV (related to C–H/C–C), 286.1 eV (related to C–OH), and 288.6 eV (related to COOH). The presence of these functional groups reflects the different carbon microenvironments in the polyaniline and graphene structures. Figure 1d displays the high-resolution spectrum of O 1s, deconvoluted into four peaks at approximately 530.7, 532.1, 533.2, and 534.1 eV, indicative of the presence of oxygen in V–O, C–O, COOH, and OH (water), respectively. The V 2p spectrum (Figure 1e) showed its V 2p_3/2_ peak at 515.2 eV. This binding energy implies the outermost surface to be composed of V­(IV or III) species rather than V­(V) as the latter would have resulted in a peak at approximately 517.0 eV.^56^ Since XPS spectra recorded for V 2p in the V_2_O_5_/rGO (VG) sample (Figure S3a–d in Supporting Information) revealed vanadium to be present as V­(V) (V 2p_3/2_: 517.0 eV), it was assumed that some of the constituents of the hydrogel media was able to reduce and stabilize vanadium in the outermost surface as V­(IV or III) rather than V­(V). Since the addition of a drop of phytic acid to the VG nanostructure solution changed the color appearance from yellow (due to V­(V), as VO_2_^+^) to greenish (typical for V­(III)), Figure S4, it was assumed that phytic acid could possibly play a role. However, the underlying mechanisms cannot be explained as phytic acid has chelating capacities stabilizing transition metal cations in specific oxidation states^57^ rather than reducing their oxidation states. Other unknown factors play a role. The XRD results revealed diffraction peaks assigned to V_2_O_5_, Figure 1b. It was therefore concluded that the change of the oxidation state of vanadium from (V) to (IV or III) only took place at the outermost surface of the VG nanostructure. This may possibly be an important factor in the enhanced electrochemical activity of the VG/HG nanocomposite reported below. Similar effects by the action of V­(IV) doping of V_2_O_**5**_ structures have been reported by Chen and co-workers.^55^ The XRD findings were supported by the Raman investigation of the VG and VG/PANi-HG materials (Figure S5). XPS revealed in addition two distinct N 1s peaks at 399.1 and 401.0 eV (Figure 1f), assigned to nitrogen in benzoide diamine and positively charged nitrogen (oxidized amine and protonated imine), respectively.^58–61^

**Figure 1:**
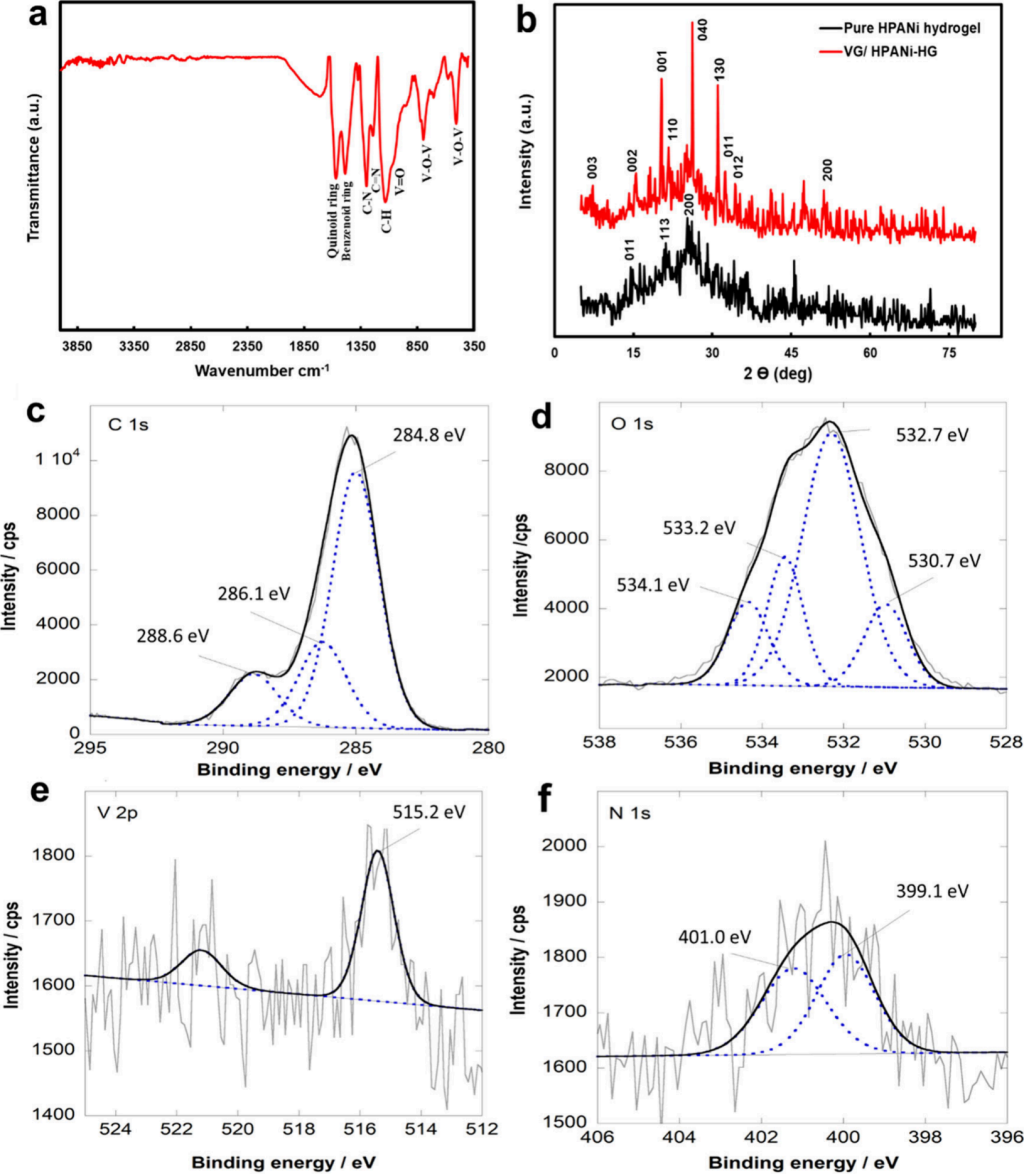
FT-IR spectrum of the VG/PANi-HG nanocomposite (a), XRD diffractograms of the pure polyaniline hydrogel (bottom) and the VG/PANi-HG composite (top) (b), and XPS spectra of VG/PANi-HG (c–f), C 1s (c), O 1s (d), V 2p (e), and N 1s (f).

SEM was employed to study the morphology of the synthesized hydrogel (Figure 2a–c) showing three-dimensional (3D) porous and foam-like structures. Higher magnifications (Figure 2b,c) show the foam-like structure of the hydrogel nanocomposite to consist of well-distributed sets of coral-shaped dendrites with average diameters of 40 nm. SEM images of the pure hydrogel are shown in Figure S6, indicating a foam-like structure. Consistent with the results of FTIR and XPS analysis, EDS confirmed the presence of carbon, nitrogen, oxygen, and vanadium (Figure S7).

### 2.2 Electrochemical Performance of the Nanocomposite

The 3D porous nanostructure of the VG/PANi-HG nanocomposite observed by means of SEM showing a large surface interfacial area available for ionic and electronic transport processes suggests an efficient electrochemical performance. Compared with the pure hydrogel, porosimetry measurements revealed an increased surface area (16.17 m^2^ g^–1^ to 18.88 m^2^ g^–1^) and increased average pore sizes (from 37.1 to 39.2 nm); see Supporting Information, Figure S8. This increase in the real surface area is anticipated to enhance the electrochemical efficiency of the nanocomposite (Figure S8).

The electrochemical properties of the synthesized VG/PANi-HG nanocomposite for potential use as an active supercapacitor material were determined using a three-electrode system employing cyclic voltammetry (CV), galvanostatic charge–discharge testing (GCDs) and AC-impedance studies (EIS). CV curves were recorded for the VG/PANi-HG, V/PANi-HG, pure PANi hydrogels, and VG in 1 M H_2_SO_4_ (scan rate 50 mV s^–1^, −0.2 to 0.8 V vs Ag/AgCl, KCl 3 M) are displayed in Figure 3a. Two redox characteristics waves (oxidation and reduction reactions) were observed for each hydrogel-based electrode material, indicative of the pseudocapacitive nature and their ability to store charge at the electrodes surface The pseudocapacitive characteristics of conducting polymers mainly originate from the redox processes (usually defined as ion doping and dedoping processes) that occur at the active sites of the electrode materials.^62^ The hydrogel structure (cf. Figure 2) enables electrolyte ion diffusion pathways to the electroactive surface of VG/PANi-HG, enhancing the electrochemical efficiency. The first redox pair was assigned to the change in oxidation state of polyaniline from leucoemeraldine (its fully reduced state) to polaronic emeraldine, and the second pair was assigned to the conversion of the polaronic emeraldine to bipolaronic pernigraniline (fully oxidized state), confirming the presence of PANi chains in the synthesized material (Figure S9). This transition was also observed as changes in visual appearance changing from whitish to bluish to greenish. At a scan rate of 50 mV s^–1^, the current density for the VG/PANi-HG electrode was remarkably higher compared to those of the other electrodes (V/PANi-HG, pure PANi-HG and VG). Detailed electrochemical studies using CV were performed at various scan rates (5 to 100 mV s^–1^) (Figure 3b). The areas under the voltammograms increased with an increased scan rate, whereas the rectangular shape of the voltammograms was almost unchanged. These observations imply rapid kinetics of the redox processes and low internal resistance of the prepared electrode materials.^24^

**Figure 2:**
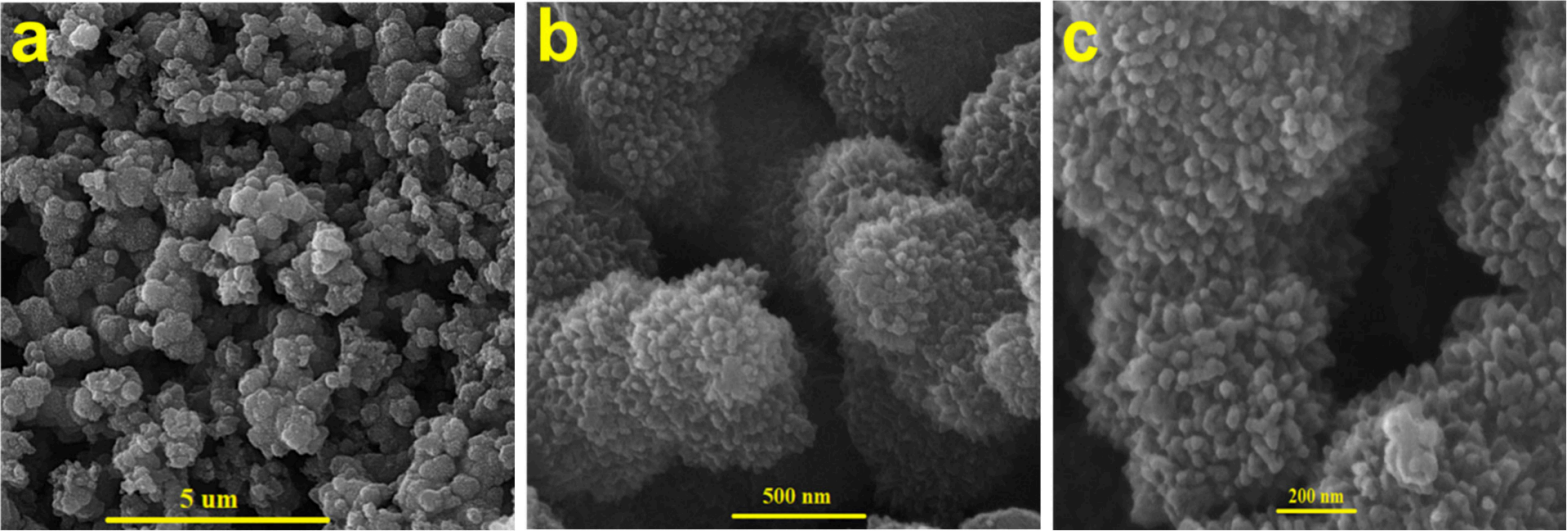
Secondary electron SEM images of VG/PANi-HG at different magnification (a–c).

The charge–discharge branches of the GCD results at various discharge currents (from 2.7 to 9 A g^–1^) are presented in Figure 3c. The plateau at a potential of around 0.6 V, mainly observed for the lower discharge currents, contributes substantially to the total specific capacitance. This behavior can be attributed to the presence of oligoanilines in the structure.^24^ The specific capacitance under different charge–discharge currents was calculated using eq 1.^63,64^

1 $$C_{s}=\frac{I\Delta t}{m\Delta V}$$

where *C*_s_ is the specific capacitance (F g^–1^), *I* is the current (A), Δ*t* is the discharge time (s), *m* is the mass of the active material (g), and Δ*V* is the applied voltage range (V).

**Figure 3:**
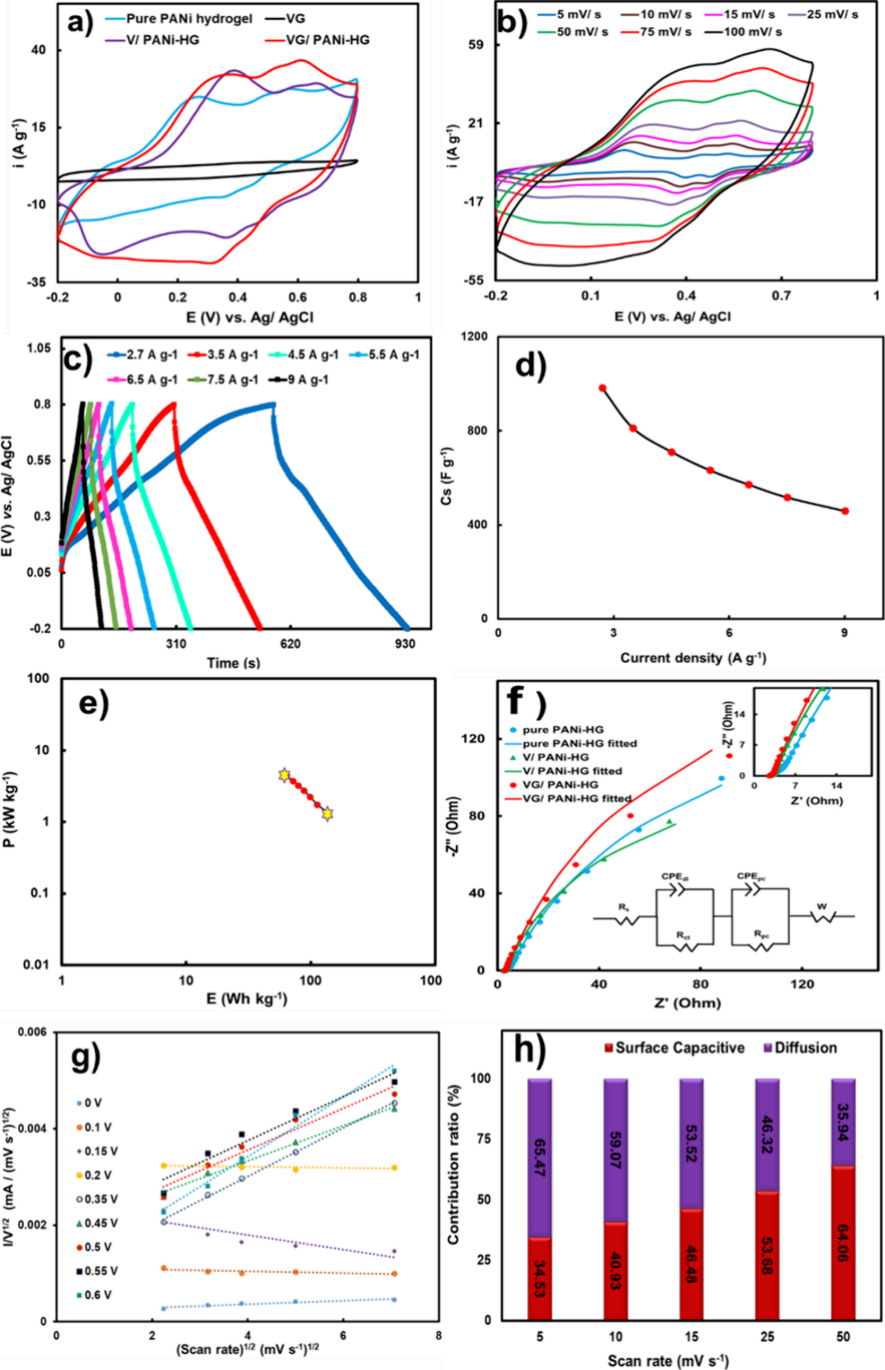
CV curves recorded for the PANi-HG, V_2_O_5_@rGO, V_2_O_5_-PANi-HG, and V_2_O_5_@rGO/PANi-HG at scan rate 50 mVs^–1^ (a), CV curves of VG/PANi-HG electrode at different scan rates (b), GCD curves of VG/PANi-HG electrode at different current densities (c), plot of specific capacitance against current density (d), Ragone plot of VG/PANi- HG electrode (e), EIS measurements for VG/PANi-HG, V/PANi, and PANi-HG electrodes (points are experimental data and lines are fitting results with proposed circuit) (f), current density versus the square root of the scan rate (g), and capacitance contribution plots (h).

The calculated specific capacitance values are plotted in Figure 3d. A specific capacitance of 982.8 F g^–1^ was determined for the VG/PANi-HG nanocomposite at a discharge current of 2.7 A g^–1^. This number is higher than most previously reported results of similar nanocomposites.^24,48,65^ The charge storage capacitance of the ternary VG/PANi-HG composite was enhanced by its morphology and the low electrical conductivity of its binder-free electrode. The presence of polyaniline on the vanadium-oxide-decorated graphene oxide facilitated rapid electrolyte penetration, which in turn reduced the charge transfer resistance and promoted ion permeation. Additionally, the carbon fiber substrate further improved the capacitance by lowering the contact resistance and increasing the conductivity at the electrolyte/electrode interface. When the current density increased to 9 A g^–1^, the specific capacitance reached almost 500 F g^–1^. This elucidates a remarkable rate capability of the nanocomposite and an ability to maintain a considerable energy density even at a high-power density (Figure S10a,b). The energy and power densities of the nanocomposite are presented in Ragone plots (Figure 3e) and calculated at different currents using eqs 2 and 3,^66^

2 $$E=\frac{0.5C_{s}V^{2}}{3.6}$$

3 $$P=\frac{E\times 3600}{t}$$

where *E* is the energy density (Wh kg^–1^), *C*_s_ the specific capacitance (F g^–1^), *V* the applied potential window (V) during the discharge process after the *iR* drop, *P* the power density (W kg^–1^), and *t* the discharging time (s). As demonstrated in Figure 3e, the VG/PANi-HG nanocomposite electrode showed a maximum power density of 4.5 kW kg^–1^ at a discharge current of 9 A g^–1^ and an energy density of 63.81 Wh kg^–1^. The VG/PANi-HG electrode showed an extremely high power density (1.35 kW kg^–1^) also when increasing the energy density to 136.5 Wh kg^–1^ at a current density of 2.7 A g^–1^, Figure 3e. This is thought to result from the distinctive hierarchical structure of the VG/PANi-HG composite, providing a highly conductive, three-dimensional network with a large accessible surface area and short ion/electron transport pathways. These structural features significantly reduce the internal resistance and facilitate rapid charge–discharge processes, thus contributing to the high-power density.

Electrochemical impedance spectroscopy (EIS) measurements were conducted to assess the electrical properties at the open circuit potential in a wide frequency range from 100 kHz to 10 mHz with an AC amplitude of 10 mV. These measurements were conducted to better understand and evaluate the performance of the polyaniline component and assess synergistic effects of polyaniline and V_2_O_5_-rGO in the VG/PANi-HG nanocomposite. These results and the electrical parameters of the VG/PANi-HG electrode are presented in Figure 3f. The Nyquist plots consisted of two distinct regions; a semicircle in the high-frequency region assigned as the charge transfer resistance (*R*_ct_) due to Faradaic reactions and a short straight line (diffusion impedance or Warburg impedance) in the low-frequency region. This line ended in a nearly vertical line (with an angle of 70°), which corroborates the considerable capacitive behavior of the electrode material.^24^ The experimental results were fitted using an equivalent circuit, shown as an inset in Figure 3f, where *R*_s_ is the electrical series resistance, CPE-1 (constant phase element) represents the electric double layer, indicating deviation from ideal capacitor, *R*_ct_ is the charge transfer resistance, and *W* describes the ion diffusion resistance at the electrode/electrolyte interface, appearing as a near vertical straight line in the low-frequency region. The presence of a second semicircle in Figure 3f indicates the pseudocapacitive contribution of the electrode materials arising from Faradaic reactions within the conducting polymer. This behavior was modeled using CPE-2 and *R*_pc_, representing pseudocapacitive charge storage and pseudocapacitive resistance, respectively. The fitting results (Table S1) indicate that the charge transfer resistance of the VG/PANi-HG nanocomposite electrode was 0.57 Ω. This is lower than the charge transfer resistance for the pure PANi-HG electrode (*R*_ct_ = 2.23 Ω) and V/PANi-HG (*R*_ct_ = 0.73 Ω), which confirms faster kinetics of the charge transfer and more reversible redox reactions in the VG/PANi-HG nanostructure. In addition, the slope of the near vertical line in the low frequency (LF) region of the nanocomposite electrode was larger compared with the PANi-HG electrode. This implies a superior capacitive behavior of the VG/PANi-HG nanocomposite. The percentages of the surface-capacitive and diffusion-controlled contributions were evaluated separately to examine the contributions of different energy storage mechanisms for the VG/PANi-HG electrode. As seen in eq 4, there is a power law relationship between the scan rate and the current density:^20,66^

4 $$i=av^{b}$$

where *v* is the scan rate (mV s^–1^), *i* is the current density (A g^–1^), and *a* and *b* are adjustable parameters. The percentage of surface-capacitive and diffusion-controlled processes can be determined by the *b*-value, which can be calculated from the slope of the curve of log­(*i*) vs log­(ν); see Figure S11. The ideal capacitive mechanism will be the dominant charge storage mechanism when *b* equals 1. The diffusion process mechanism for determining the capacitance *b* will be 0.5. However, even *b*-values between 0.5 and 1 show that the contribution of both mechanisms is important. From Figure S11, the *b* value for VG/PAni-HG was approximately 0.72. This confirms that the electrode process is mediated by the synergistic contribution of the capacitive response and diffusion-limited currents. Using eq 5, the percentage of contribution for diffusion- and capacitive-controlled processes can be calculated quantitatively:^20,66^

5 $$i\left(V\right)=k_{1}v+k_{2}v^{1/2}$$

The current contributions from the surface-capacitive (double layer) and diffusion-controlled mechanisms of charge storage are denoted by *k*_1_ν and *k*_2_ν^1/2^, respectively (Figure 3g). The graphical representation of the contribution of each capacitance mechanism at various potential scan rates is presented in Figure 3h. The figure also demonstrates that the diffusion-controlled contributions dominate over the surface-capacitive contribution at 65.5% at lower scan rates but *i* will gradually decrease to ≈36% at higher scan rates.

To assess the operational capabilities of the synthesized VG/PANi-HG electrode in energy storage devices, an all-solid-state asymmetric supercapacitor was designed and assembled, as illustrated in Figure 4a. The positive electrode in the asymmetric device was the VG/PANi-HG electrode with graphene oxide (GO) brushed onto a carbon fiber substrate (GO/CF) electrode as the negative electrode. Polyvinyl alcohol gel containing 1 M H_2_SO_4_ was used as electrolyte. The electrochemical investigations, Figure 4b (*i*–*V* curves at various voltage scan rates, from 5 to 100 mV s^–1^), revealed a very high capacitive performance of the asymmetric device in the voltage range of 0.0 to 1.6 V. By increasing the scan rate from 5 to 100 mV s^–1^, the capacitive behavior was maintained and the shape of the voltammograms remained relatively unchanged. The specific capacitance of the asymmetric device was almost 195.31 F g^–1^ at a scan rate of 5 mV s^–1^. As illustrated in Figure 4c, the specific capacitance slightly decreased with an increased scan rate. These findings support the very high capability of the device even at high scan rates. Galvanostatic charge–discharge tests for the ASC device were carried out at different currents (1.08 to 4.34 A g^–1^) (Figure 4d). A specific capacitance of 209.76 F g^–1^ was obtained at a current density of 1.08 A g^–1^ (Figure 4e), which implies fast and easy penetration of the electrolyte into the porous structure of the hydrogel nanocomposite. The energy and power densities of the ASC device were plotted (Ragone plots) and calculated for different current densities using eqs 3 and 4 (Figure 4f). This asymmetric device showed outstanding power and energy densities of almost 2 kW kg^–1^ and 74.58 Wh kg^–1^, respectively. Compared to similar reported studies, these findings confirm the excellent performance of the ASC supercapacitor.^38,50,67–74^

Cycle stability is another critical parameter to assess the supercapacitor properties. These results are presented in Figure 4g and show that almost 94% of the initial capacitance of the ASC device remained after 10 000 successive cycles at a high scan rate of 125 mV s^–1^, emphasizing its long-term operational stability. The observed decrease in hydrogel capacitance before 2000 cycles is attributed to the loss of fine particles, which possess weak and unstable bonds within the hydrogel structure. Prolonged electrochemical cycling is believed to lead to enhanced penetration of electrolyte ions into the deeper regions of the hydrogel, promoting chain movements of the polymer, leading to improved electrochemical behavior and capacitance. To inspect the practical stability of the ASC device under the operational conditions, EIS measurements for the ASC device before and after the cyclic stability test show only slight variations in charge transfer resistance over 10,000 successive cycles, Figure 4h. Using an appropriate Randles model (in which CPE_dl_ was used instead of *C*_dl_ to model the nonideality of double layer capacitor) to fit the experimental results (shown in Supporting Information, Table S2), it was clear that *R*_ct_ slightly increased from 4.71 to 5.85 Ω. The electrode material microstructure was also examined using SEM and FTIR analyses after 10 000 cycles. These analyses, confirming the high stability of this supercapacitor, are presented in Figure S12. These results verify the considerable operational stability of the VG/PANi-HG based supercapacitor.

**Figure 4:**
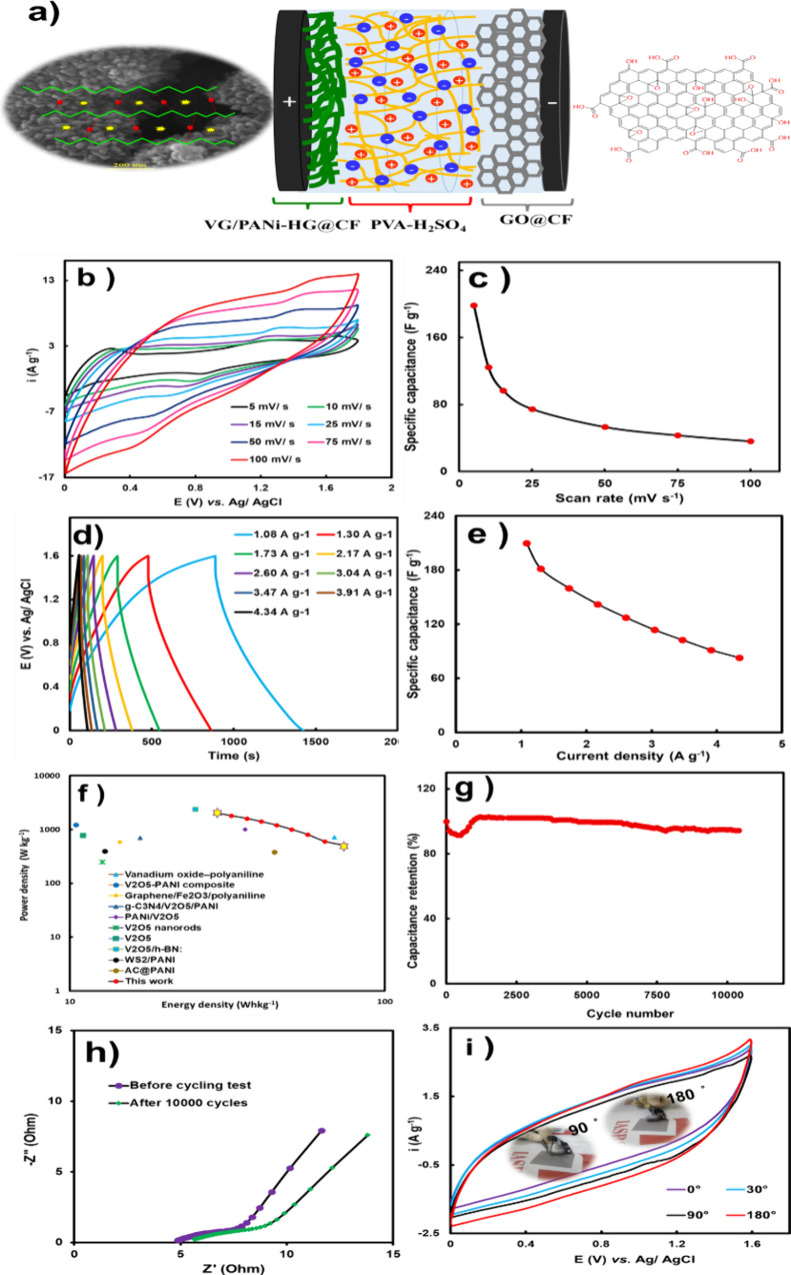
Schematic description of the VG/PANi-HG//rGO ASC device (a), CV curves of ASC device at different scan rates (b), specific capacitance plot of ASC device at different scan rates (c), GCD curves of ASC device at different current densities (d), specific capacitance plot of ASC device at different current densities (e), comparison of VG-PANi-HG based ASC device Ragone plots with previously reported similar works (f), cyclic stability of the ASC device at a scan rate of 125 mV s^–1^ for 10 000 cycles (g), EIS plot of the as-prepared asymmetric supercapacitor system before and after 10 000 successive charge–discharge cycles (h), and CV curves of ASC device at 0°, 30°, 90° and 180° bending angles (i).

Flexible electronics are important drivers of technological progress, for instance, the use of wearable electronics connected to modern personalized healthcare. This requires flexible energy storage devices such as flexible supercapacitors.^75–78^ Such flexible supercapacitors, which utilize hydrogel-based electrodes and electrolytes, demonstrate excellent mechanical stability along with key energy storage capabilities, including high power and energy densities and outstanding cycling stability.^79–82^ To validate the flexibility of the assembled ASC hydrogel-based supercapacitor, its voltammetric behavior was investigated under different bending angles (0°, 30°, 90°, and 180°) at a scan rate of 50 mV s^–1^. The results are presented in Figure 4i. Surprisingly, the curves with and without bending showed nonsignificant differences, confirming the ample flexibility of the ASC during electrochemical operation. To evaluate the fatigue behavior, the electrochemical behavior of the electrode at a bending angle of 90° after successive mechanical bending (10 and 100 times) was investigated by means of cyclic voltammetry at a scan rate of 50 mV s^–1^. The results show very good flexibility of this supercapacitor (Figure S13). Essential energy storage parameters of the synthesized hydrogel-based supercapacitor are compiled in Table 1 and compared to literature findings of similar supercapacitors. The ASC device including the synthesized VG/PANi-HG nanocomposite as electrode, showed remarkedly better results compared to literature findings.^24,38,40,48,70,71,83^ From this follows that the VG/PANi-HG nanocomposite is a promising future electrode material for energy storage devices, especially for flexible electronic applications.

**Table 1:** Critical Energy Storage Parameters of the Assembled Hydrogel-Based Supercapacitor of This Study Compared to Literature Findings for Similar Supercapacitors

electrode material	electrolyte	potential window (V)	specific capacitance (F g^–1^)	cycling stability (%)	ref
PANi/V_2_O_5_ hybrid	1 M H_2_SO_4_	–0.3 V to 0.7 V	498 (1 A g^–1^)	94% (10 000 cycles)	^70^
V_3_O_7_-rGO-PANi (HCl dopant)	1 M H_2_SO_4_	–0.1 V to 1 V	597 (0.2 A g^–1^)	94% (2500 cycles)	^48^
Vanadium oxide-PANi composite nanowire	5 M LiCl	–0.9 V to 0.7 V	443 (0.5 mA cm^–2^)	92% (5000 cycles)	^71^
Hydrogel of ultrathin pure PANi nanofibers	1 M H_2_SO_4_	–0.2 V to 0.8 V	636 (2 A g^–1^)	83.3% (10 000 cycles)	^84^
PANi/graphene hydrogels	1 M H_2_SO_4_	–0.2 to 0.6 V	610 (1 A g^–1^)	94.4% (1000 cycles)	^40^
PANi (PANI) modified oriented graphene hydrogel (OGH)	H_2_SO_4_-PVA	0 to 0.8 V	530 (1 A g^–1^)	80% (10 000 cycles)	^83^
PANi/graphene/Fe_2_O_3_ composites hydrogel	1 M H_2_SO_4_	–0.2 V to 0.8 V	1124 (0.25 A g^–1^)	82% (10 000 cycles)	^38^
Flexible graphene/polyaniline nanofiber	1 M H_2_SO_4_	–0.2 to 0.8 V	210 (0.3 A g^–1^)	71% (800 cycles)	^85^
Polyaniline/rGO aerogel	1 M H_2_SO_4_	–0.2 to 0.8 V	432 (1 A g^–1^)	85% (10 000 cycles)	^86^
SWCNT/PANI	1 M H_2_SO_4_	0 V to 1 V	541 (20 mV s^–1^)	80% (5000)	^87^
PANi-GP	0.5 M H_2_SO_4_	–0.2 to 0.8 V	176 mF cm^–2^ (0.2 mA cm^–2^)	74.8% (after 500 bending)	^88^
V_2_O_5_/rGO/PANi hydrogel (VG/PANi HG)	1 M H_2_SO_4_	–0.2 V to 0.8 V	982 (2.7 A g^–1^)	94% (10 000 cycles)	This study

## 3 Conclusions

A novel electrode material of hydrogel-based nanocomposite of polyaniline and V_2_O_5_/rGO was designed and synthesized for intended use in a supercapacitor device. The electrode structure and composition as well as its electrochemical properties were investigated using a multitude of different analytical and electrochemical techniques. The VG/PANi-HG electrode exhibited a very good specific capacitance of 982 F g^–1^ at a discharge current of 2.7 A g^–1^. A flexible all-solid-state asymmetric device, based on the VG/PANi-HG electrode as the positive electrode, was integrated using the PVA hydrogel as the electrolyte. This asymmetric device showed outstanding power and energy densities of almost 2 kW kg^–1^ and 74.58 Wh kg^–1^, respectively, as well as a long-term operational stability. The VG/PANi-HG electrode demonstrated exceptional performance, exhibiting an outstanding cyclic life stability of 94% after 10 000 cycles. Its high flexibility at various bending angles and remarkable operational stability, even at a 90° bending angle, further indicate its suitability as a promising candidate as an electrode material in flexible supercapacitors.

## 4 Experimental Section

### 4.1 Materials

Ammonium metavanadate, phytic acid (50% w/w), graphite flake, and aniline monomer were purchased from Sigma-Aldrich, and ammonium persulfate (98%) and sulfuric acid (98% w/v) were purchased from Merck. Deionized water (18 MΩ cm) was used throughout all experiments. Other chemicals were of analytical grade and used as received.

### 4.2 Preparation of V_2_O_5_

For V_2_O_5_ synthesis, 70 mL of a 0.1 M ammonium metavanadate solution was prepared in deionized water. The pH of the solution was adjusted to 2.0 by adding an appropriate amount of diluted acid solution and measured using a Metrohm pH-meter (model 780). The resulting solution was transferred to a 100 mL hydrothermal autoclave and kept at a temperature of 150 °C for 18 h. After the autoclave was cooled, the precipitate was washed with absolute ethanol and deionized water and dried at 80 °C for 3 h. Finally, the precipitate was calcined at 400 °C for 3 h.

### 4.3 Preparation of V_2_O_5_-rGO (VG) Nanocomposite

Graphene oxide was prepared using a modified Hammers’ method from graphite oxide.^89–91^ In order to synthesis the V_2_O_5_-rGO composite, 0.21 g of rGO was well dispersed in 70 mL deionized water sonicated using an ultrasonic water bath for 2 h (solution A) after which 0.42 g of the as-prepared V_2_O_5_ was added and stirred for 1 h. Five mL of hydrogen peroxide (H_2_O_2_, 33% w/v) was added to the resulting mixture and stirred for another 2 h. Finally, the resulting mixture was placed in an autoclave under hydrothermal conditions (150 °C) for 15 h. The final precipitate was the V_2_O_5_-rGO (VG) nanocomposite.

### 4.4 Preparation of V_2_O_5_-rGO/PANi Hydrogel (VGO/PANi-HG)

1.842 mL portion of a 50% phytic acid solution was sonicated in a water bath with 0.485 mL of aniline monomer for 30 min, followed by stirring (700 rpm) for 12 h (solution 1). At the same time, a 10 mg mL^–1^ suspension of VG was prepared in water and phytic acid (2 mL) using vigorous stirring (1900 rpm) (solution 2) for 60 min. This was followed by the slow addition of solution 2 to solution 1 that was ultrasonically mixed for at least 1 h. The resulting mixture was then centrifuged (Spectrafuge 6C, Labnet International Inc.) for 5 min at 1000 rpm to separate bulky and large particles. In the next stage, a piece of carbon fiber substrate (1 cm × 1 cm) was placed in the homogenized solution (solution 3) and positioned in an ice bath. In parallel, a 2 mL solution of deionized water and phytic acid solution containing 0.286 g of ammonium persulfate (APS) (solution 4) was prepared. Solution 4 was then slowly added to solution 3, until the polymerization reaction took place during 12 h in an ice bath. The resulting electrodes were washed with deionized water and dried at 60 °C for 3 h. The mass of the deposited material per surface area was 1 mg cm^–2^. Figure S14 shows a schematic illustration of the hydrogel electrode synthesis setup.

### 4.5 Characterization

The synthesized nanocomposite was characterized in terms of presence of functional surface groups by means of transmission Fourier transform infrared spectroscopy (FTIR). Composition and oxidation state of elements at the outermost surface were assessed by means of X-ray photoelectron spectroscopy (XPS), the crystallinity and composition of phases by means of X-ray diffraction (XRD), and surface morphology and bulk elemental composition by means of scanning electron microscopy with energy dispersive spectroscopy (SEM/EDS).

FTIR measurements were performed using a Vector-22, Bruker instrument, acquiring spectra with a 2 cm^–1^ resolution. Transmission spectra was obtained on pellets of the powder mixed with KBr. XPS overview and detailed scans of V 2p, O 1s, N 1s, and C 1s (pass energy of 20 eV) were acquired using a Kratos Ultra-DLD spectrometer, Kratos Analytical. Measurements were recorded with a 150 W source power, and energy correction was made versus the C 1s peak of adventitious carbon set at 284.8 eV. XRD spectra were recorded using a Bruker instrument, D8-advanced using Cu Kα1 radiation (λ = 1.5406 Å). SEM and EDS investigations were performed using a MIRA3 TESCAN-XMU microscope equipped with an Oxford EDS detector.

### 4.6 Electrochemical Performance

An Autolab model PGSTAT101 potentiostat/galvanostat (The Netherlands) and a Zahner/Zennium instrument (Germany) were used for all electrochemical testing. An Ag/AgCl electrode (as a reference electrode) and platinum-wire electrode (as an auxiliary electrode) manufactured by Metrohm were employed in addition to PANi-HG and VG/PANi-HG electrodes as working electrodes in three-electrode setup. For the two-electrode investigations, the VG/PANi-HG electrode was used as the positive electrode, and graphene oxide brushed carbon fiber (GO/CF) was used as the negative electrode. Mass balance for both the negative and the positive electrodes was carried out according to eq 6:

6 $$\frac{m_{+}}{m_{-}}=\frac{C_{-}\Delta V_{-}}{C_{+}\Delta V_{+}}$$

where *m* indicates the mass of the active material, Δ*V* the potential window, and *C* the specific capacitance of the positive (+) and the negative (−) electrodes, respectively. All electrochemical experiments were performed at ambient laboratory temperature.

## Supplementary Material







## Notes

Supplementary Figures S1–S14, mass balance calculations, and desciption of videos (PDF)
Movie S1 showing electrochemical behavior of prepared asymmetric supercapacitor against 90° bending (MP4)
Movie S2 showing electrochemical behavior of prepared asymmetric supercapacitor against 180° bending (MP4)
