# Supplementary material for: A Flexible All-Solid-State Asymmetric Supercapacitor Based on a Nanocomposite of Vanadium Oxide/Graphene and Polyaniline Hydrogel with Excellent Operational Stability and Energy Density
Source: ACS Omega. 2025 Sep 15;10(41):47973–84. doi: 10.1021/acsomega.5c03646 (PMC12547519; doi:10.1021/acsomega.5c03646)
Supplement: Supplementary file 1 [file ao5c03646_si_001.pdf]

SUPPORTING INFORMATION

A flexible all-solid-state asymmetric supercapacitor based on a nanocomposite of vanadium oxide/graphene and polyaniline hydrogel with excellent operational stability and energy density

*Mohammad Barazandeh[a], Sayed Habib Kazemi[*a], Farzad Roohi[a], Dawod S. Haydar[a,b], Inger Odnevall[*c]*

[a] *Department of Chemistry, Institute for Advanced Studies in Basic Sciences (IASBS), 45137-66731, Zanjan, Iran*

[b] *Department of Chemistry, College of Science, Salahaddin University-Erbil, Erbil,Kurdistan region, Iraq*

[c] *KTH Royal Institute of Technology, Dep. Chemistry, Div. Surface and Corrosion Science, SE-100 44 Stockholm, Sweden*

**Corresponding author(s): Sayed Habib Kazemi (habibkazemi@iasbs.ac.ir), Inger Odnevall (ingero@kth.se)*

Part one: ADDITIONAL CHARACTERIZATION

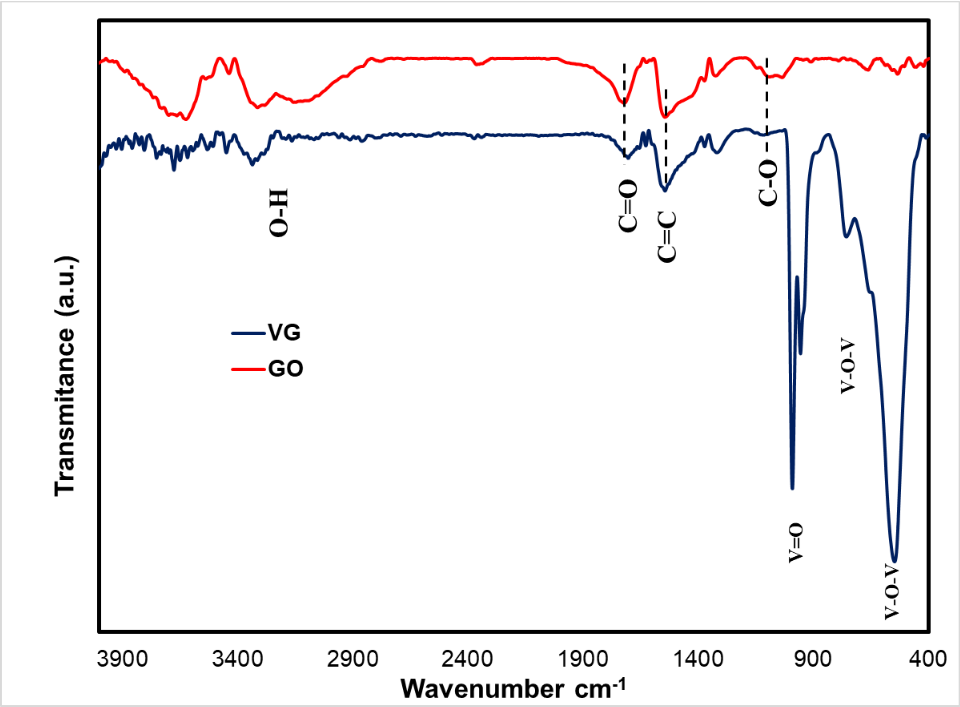

Figure S1. FTIR analysis for graphene oxide (GO) and graphene oxide/vanadium oxide (VG)

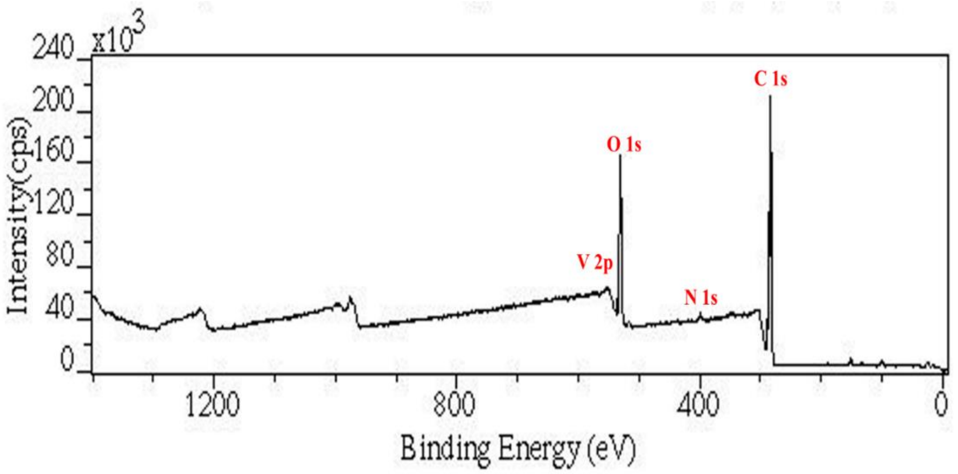

Figure S2. XPS survey spectrum of VG/PANi-HG showing the characteristic bands of carbon (C1s), oxygen (O1s), nitrogen (N1s) and vanadium (V2p).

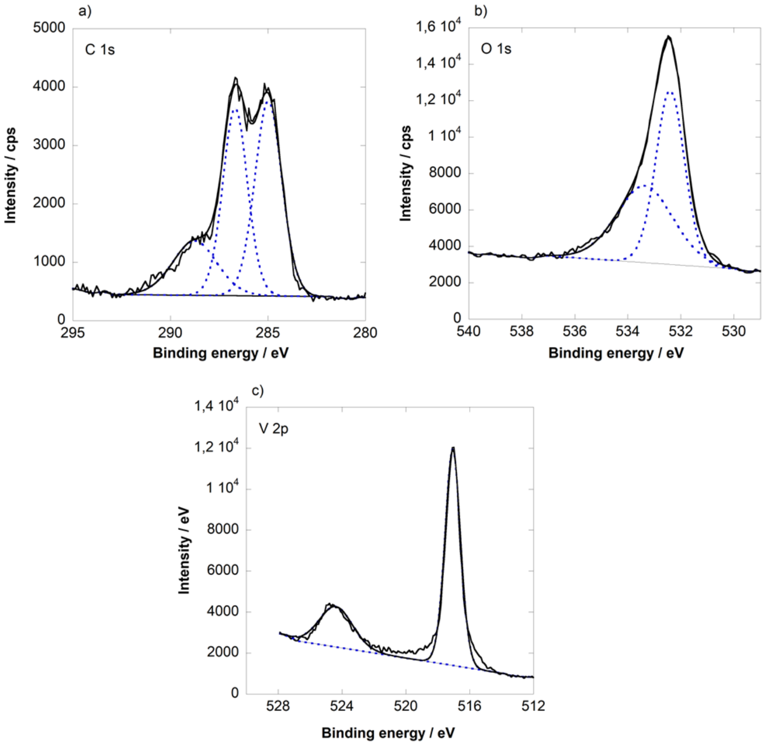

Figure S3. Detailed XPS spectra recorded for C 1s (a), O 1s (b) and V 2p (c) for the V_2O_5/rGO (VG) sample.

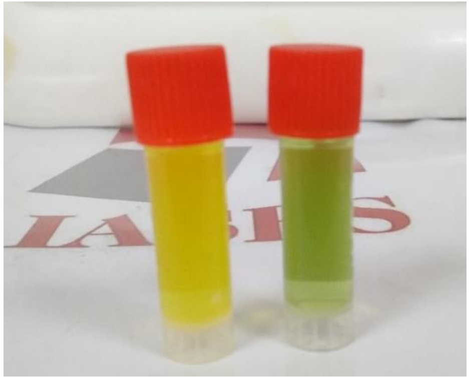

Figure S4. Optical photograph showing changes in color of a solution of the VG nanostructure from yellow (left tube) to green (right tube) when adding a drop of phytic acid to the VG nanostructure solution.

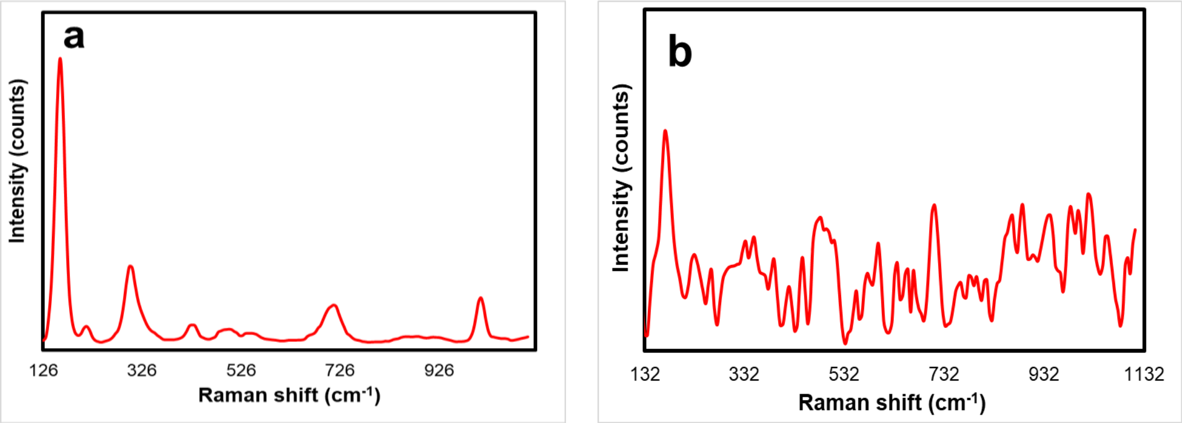

Figure S5. Raman spectra of VG (a) and VG/ PANi-HG (b).

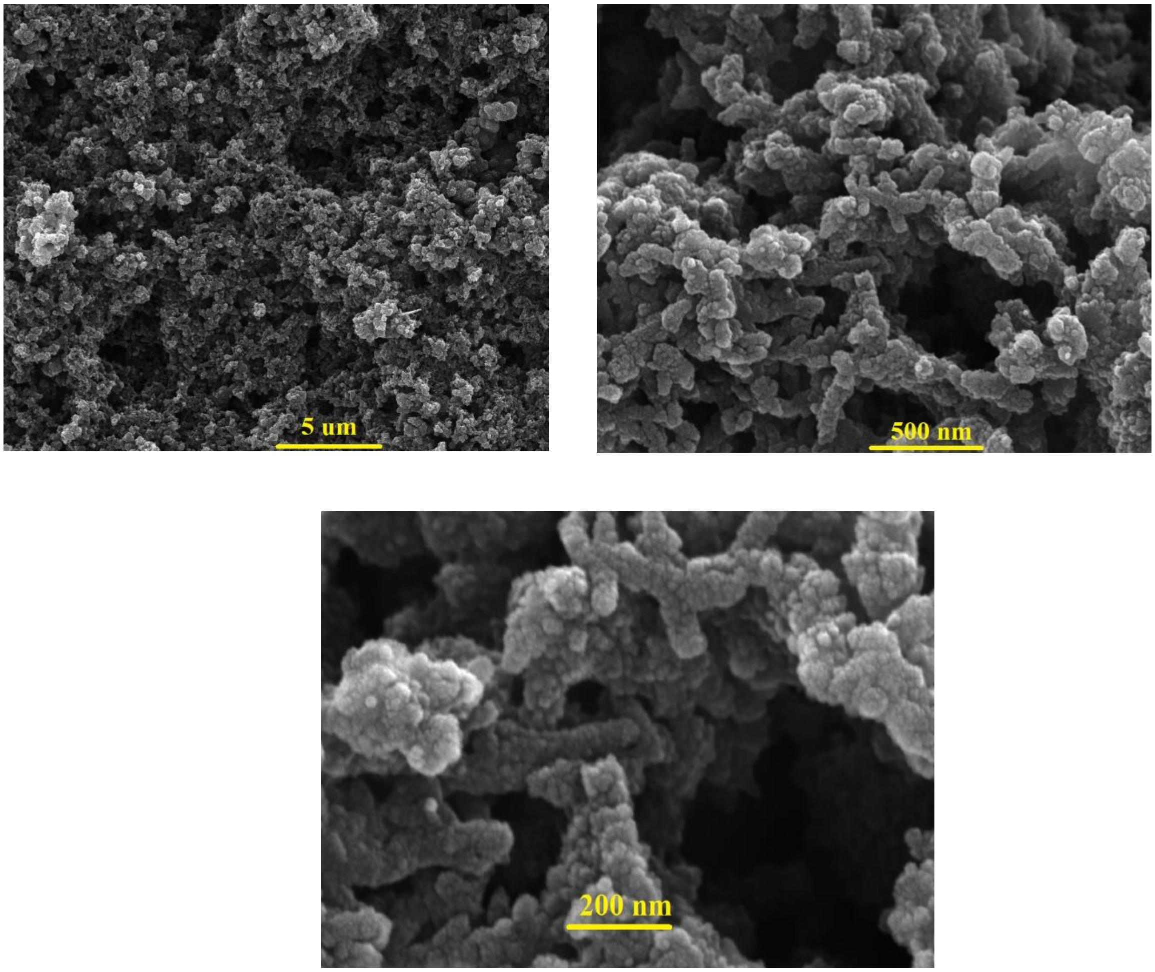

Figure S6. SEM images of PANi-HG at different magnification after 10,000 cycles.

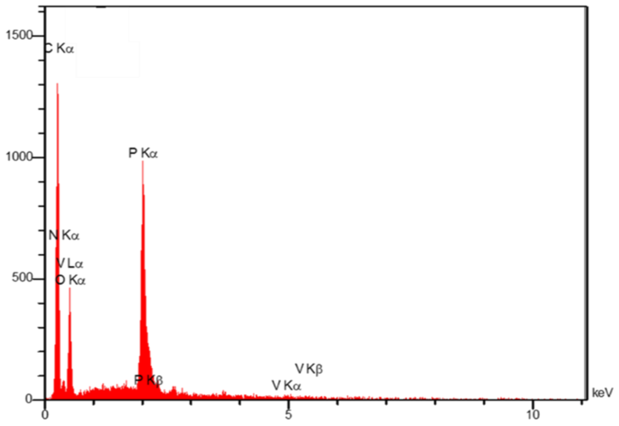

Figure S7. EDX analysis of the nanocomposite showing the presence of carbon, nitrogen, oxygen, and vanadium.

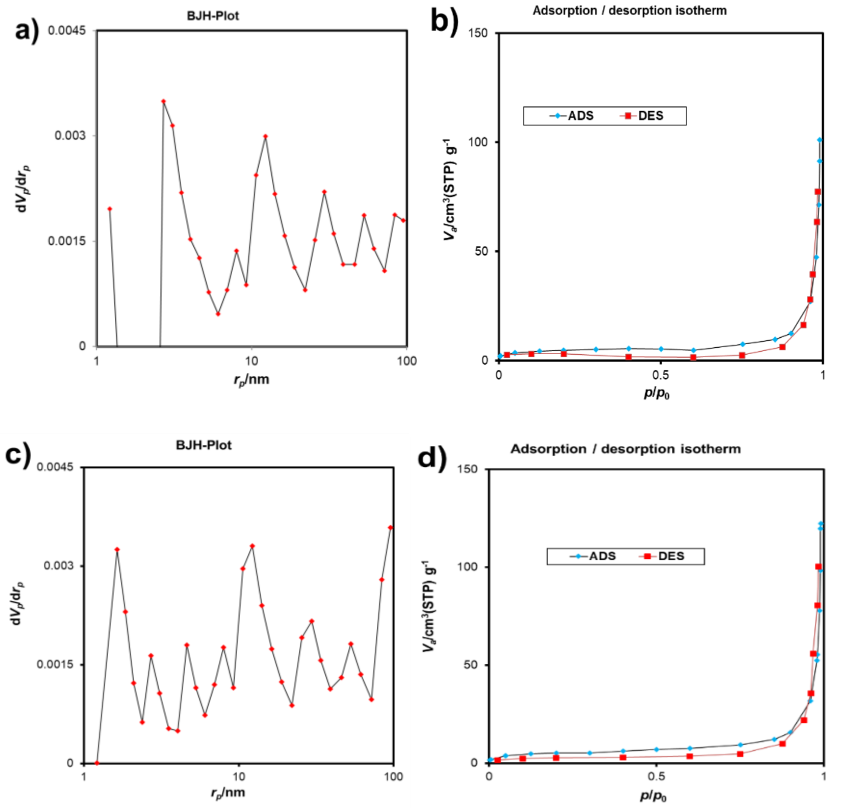

Figure S8. BET surface area, and pore size distribution of pure HPANi (a, b) and VG/ PANi-HG (c,d)

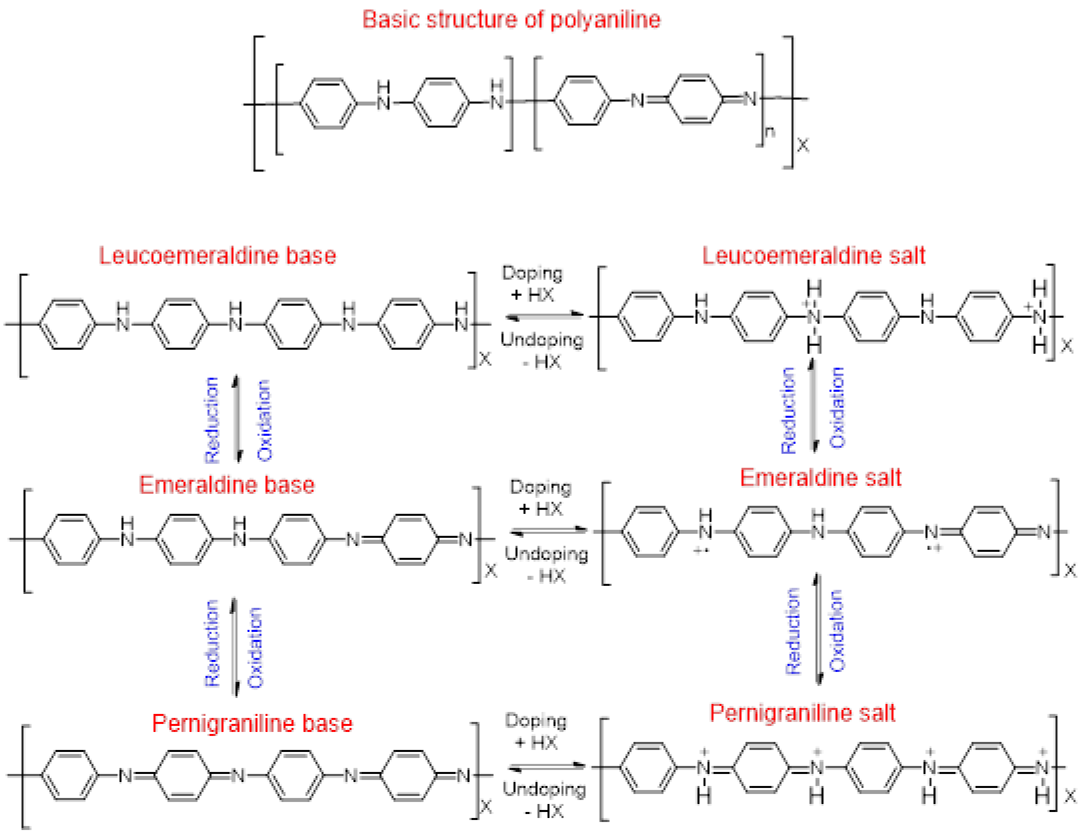

Figure S9. Proposed mechanism of electrochemical reactions.

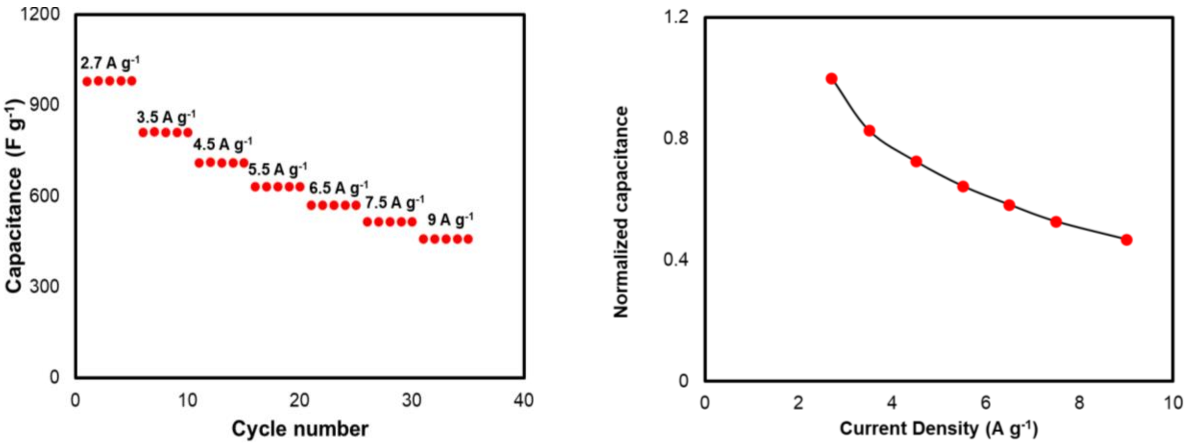

Figure S10. Normalized rate performance graphs (based on active mass) for VG/PANi-HG electrode

Table S1. Electrical parameters from fitting of electrochemical impedance spectroscopy studies of pure PANi hydrogel and its composites

Samples	R_s (Ohm)	R_{ct} (Ohm)	W (Ohm)
PANi-HG	2.73	2.23	0.31
V/ PANi-HG	2.64	0.73	0.16
VG/ PANi-HG	2.55	0.57	0.11

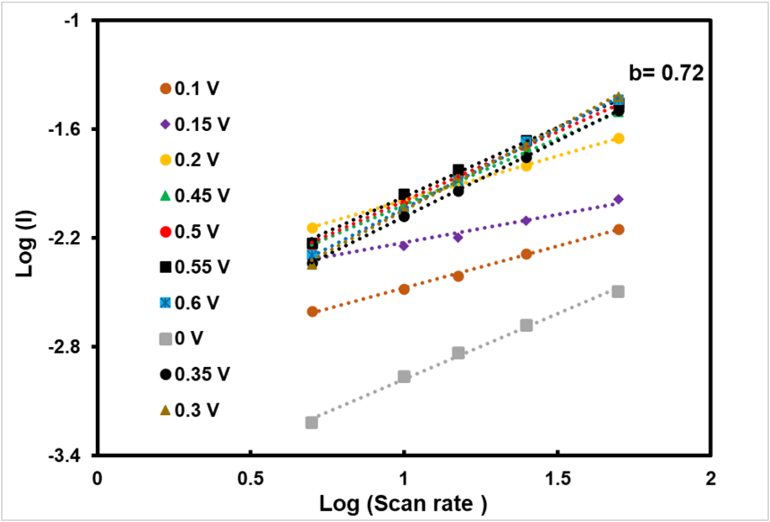

Figure S11. Log(i) against log(υ) plots for VG/PANi-HG electrode

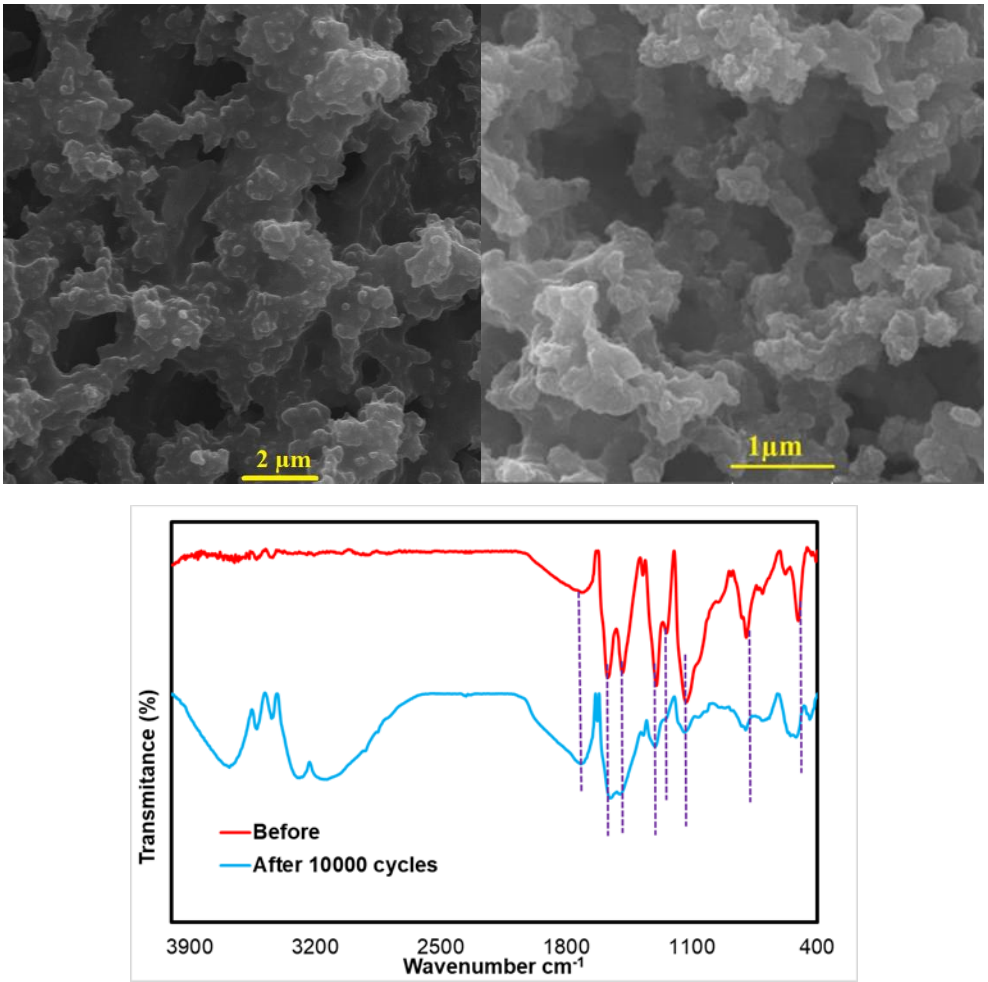

Figure S12. Analysis results for operational stability of the VG/PANi-HG based supercapacitor: SEM images: before (left) and after (right) 10,000 cycles.
FT-IR spectra: before (red spectrum) and after (blue spectrum) 10,000 cycles.

Table S2. Electrical parameters of VG/PANi-HG based supercapacitor using a modified Randles circuit, before and after 10,000 cycles (stability test)

Samples	R_s (Ohm)	R_{ct} (Ohm)	W (Ohm)
**Before stability**	4.64	4.71	0.11
**After stability**	5.26	5.85	0.11

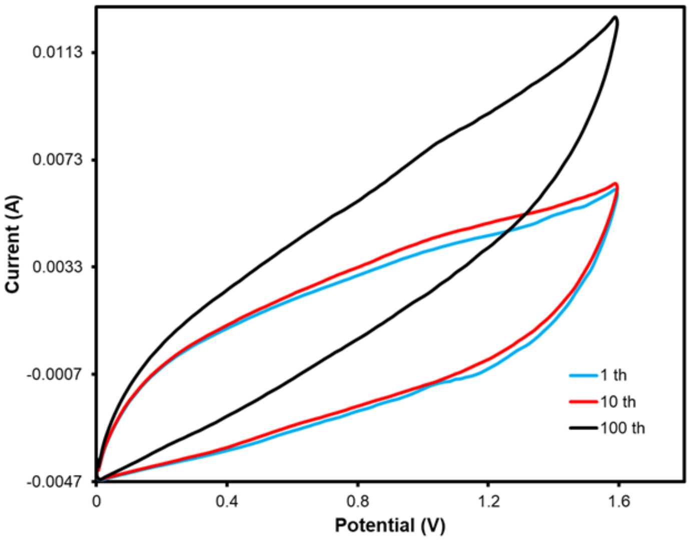

Figure S13. Electrochemical behavior of the electrode at a bending angle of 90 degrees after successive 10 and 100 times of mechanical bending

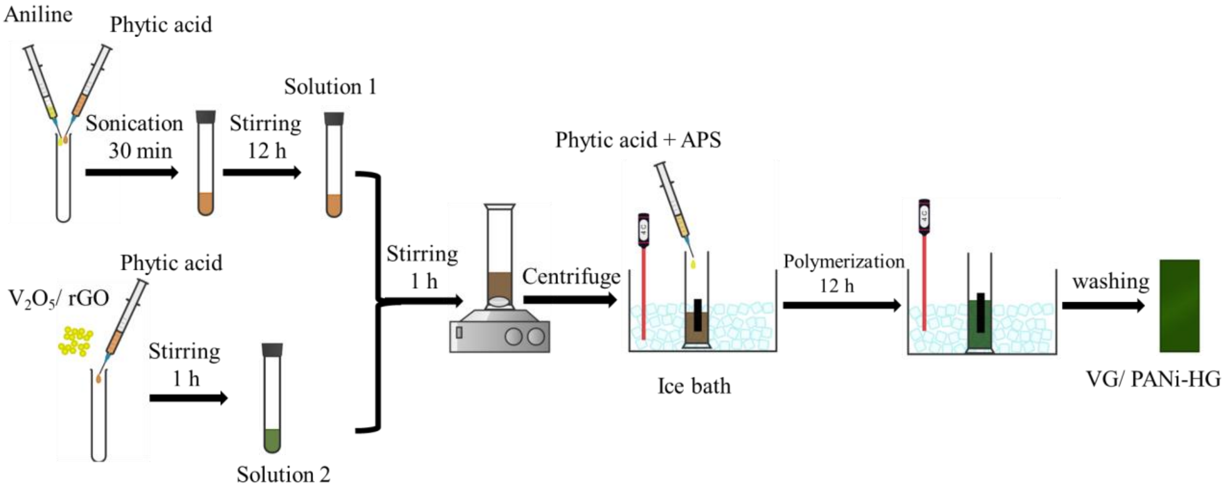

Figure S14. Schematic representation of V_2O_5-rGO/PANi hydrogel (VG-PANi HG) synthesis

Part 2: MASS BALANCE DETAILS FOR ASSYMETRIC DEVICE FABRICATION

The VG/PANi-HG electrode was made during the production of the nanocomposite, and the weight of the active material was determined using a precise balance of 1.2 mg. The weight of the rGO@CF electrode in the two-electrode system was balanced using the following formula:

$$\frac{m_+}{m_-} = \frac{C_-\Delta V_-}{C_+\Delta V_+}$$

$$\frac{m_+}{m_-} = 0.254$$

where *m* indicates the mass of the active material, ΔV indicates the potential window, and *C* indicates the specific capacitance of the positive (+) and the negative (-) electrodes.

Part 3: SUPPLEMENTARY MOVIES

Movie S1. Electrochemical behavior of prepared asymmetric supercapacitor against 90° bending

Movie S2. Electrochemical behavior of prepared asymmetric supercapacitor against 180° bending
